# Molecular Mechanisms of Microvascular Obstruction and Dysfunction in Percutaneous Coronary Interventions: From Pathophysiology to Therapeutics—A Comprehensive Review

**DOI:** 10.3390/ijms26146835

**Published:** 2025-07-16

**Authors:** Andre M. Nicolau, Pedro G. Silva, Hernan Patricio G. Mejía, Juan F. Granada, Grzegorz L. Kaluza, Daniel Burkhoff, Thiago Abizaid, Brunna Pileggi, Antônio F. D. Freire, Roger R. Godinho, Carlos M. Campos, Fabio S. de Brito, Alexandre Abizaid, Pedro H. C. Melo

**Affiliations:** 1 Instituto do Coração, Faculdade de Medicina, Universidade de São Paulo (InCor/HCFMUSP), São Paulo 05403-900, Brazil; 2 Cardiovascular Research Foundation, New York, NY 10019, USA

**Keywords:** coronary microvascular obstruction, microvascular dysfunction, no-reflow phenomenon, reperfusion injury

## Abstract

Coronary microvascular obstruction and dysfunction (CMVO) frequently arise following primary percutaneous coronary intervention (PCI), particularly in individuals with myocardial infarction. Despite the restoration of epicardial blood flow, microvascular perfusion might still be compromised, resulting in negative clinical outcomes. CMVO is a complex condition resulting from a combination of ischemia, distal thrombotic embolization, reperfusion injury, and individual susceptibilities such as inflammation and endothelial dysfunction. The pathophysiological features of this condition include microvascular spasm, endothelial swelling, capillary plugging by leukocytes and platelets, and oxidative stress. Traditional angiographic assessments, such as Thrombolysis in Myocardial Infarction (TIMI) flow grade and myocardial blush grade, have limited sensitivity. Cardiac magnetic resonance imaging (CMR) stands as the gold standard for identifying CMVO, while the index of microvascular resistance (IMR) is a promising invasive option. Treatment approaches involve powerful antiplatelet drugs, anticoagulants, and supersaturated oxygen, yet no treatment has been definitively shown to reverse established CMVO. CMVO remains a significant therapeutic challenge in coronary artery disease management. Enhancing the comprehension of its core mechanisms is vital for the development of more effective and personalized treatment strategies.

## 1. Introduction

Despite significant advancements in the management of ST-elevation myocardial infarction (STEMI), particularly with the widespread adoption of primary percutaneous coronary intervention (PCI), reduction in door-to-balloon times, and improvements in infarct-related artery patency, STEMI continues to be linked to elevated mortality and morbidity [1,2]. Often, restoration of epicardial coronary patency is not accompanied by adequate microcirculatory perfusion. The phenomenon of inadequate myocardial perfusion after temporary coronary occlusion without angiographic evidence of mechanical vessel obstruction is traditionally known as “no-reflow”. Its pathophysiological substrate consists of coronary microvascular obstruction and dysfunction (CMVO), and these terms are commonly used interchangeably [3].

The no-reflow phenomenon is very common, with its prevalence varying widely depending on the definition and method used. It is identified in about 10–50% of STEMI patients and is a strong predictor of adverse events [4,5,6,7]. This was demonstrated in several studies, including an individual, patient-level meta-analysis that included 1688 patients from 7 randomized clinical trials, which were assessed by cardiac magnetic resonance (CMR) within 7 days following primary PCI. A strong and independent link was found between CMVO and mortality over one year, as well as hospitalizations for heart failure. Numerically, in an adjusted model, every 1% absolute increase in CMVO extent was independently associated with a 14% relative increase in 1-year all-cause mortality and an 8% increase in 1-year heart failure (HF) hospitalization [5].

While the term “no-reflow” refers to blood flow stasis or CMVO associated with PCI in both stable chronic coronary syndrome and acute coronary syndrome patients, it more accurately describes an ischemia–reperfusion syndrome that occurs following a coronary artery blockage and subsequent reopening. Thus, the blood flow stasis recognized after elective PCI, although it may not fully meet the criteria for the no-reflow phenomenon, the results from plaque disruption due to manipulation, platelet activation, and inflammation response. In non-acute myocardial infarction (AMI) scenarios, CMVO occurs during or immediately after PCI, resulting in myocardial ischemia. It is also strongly associated with higher rates of myocardial injury and infarction [8]. Regarding primary PCI in STEMI patients, this phenomenon is preceded by ischemic cell injury and is also a consequence of reperfusion injury. It serves as an independent predictor of adverse clinical outcomes following AMI, regardless of infarct size, and is associated with an increase in all-cause mortality and heart failure hospitalization [5,9].

Despite the advances in protocols and percutaneous devices for the treatment of myocardial infarction, STEMI-related mortality has remained stable over the years [10,11]. Prevention and treatment of CMVO is a promising target that may contribute to lowering these rates. At this point, despite extensive efforts, a definitive therapy to consistently prevent or reverse CMVO has not yet been established. Therefore, a deeper understanding of pathophysiological pathways and possible targets for therapy remains paramount; it is the objective of this review to highlight such targets.

## 2. Pathophysiology

**Figure 1:**
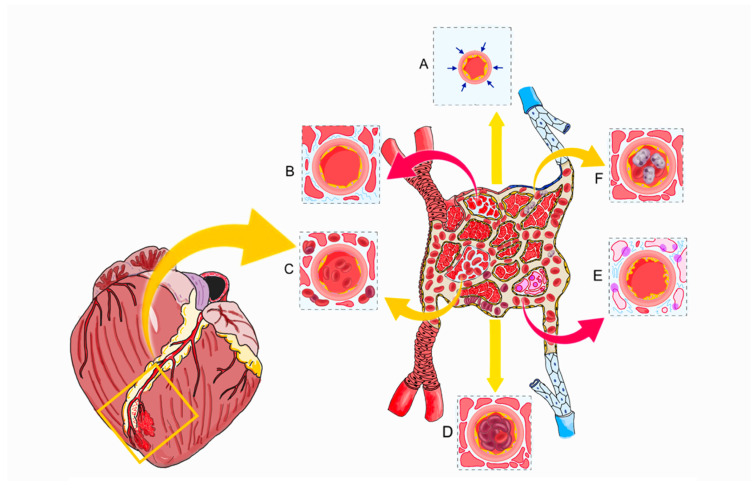
**Pathophysiological mechanisms of microvascular obstruction and coronary no-reflow**. Pathophysiological processes occurring at both the level of the epicardial coronary arteries and the coronary microcirculation. (**A**). Spasm; (**B**). myocardial swelling; (**C**). intramyocardial hemorrhage; (**D**). distal embolization; (**E**). endothelial damage and myocardial necrosis; (**F**). leukocyte plug.

CMVO is the main underlying cause of coronary no-reflow. It arises from a complex interaction of factors impacting myocardial microcirculation, such as myocardial ischemia, distal embolization, reperfusion injury, and individual susceptibility (Figure 1).

***Microcirculation:*** The coronary microcirculation encompasses vessels <200 µm in diameter and are, therefore, invisible on standard coronary angiography. Characterized by a proportionally thick smooth muscle wall, arterioles serve as the primary resistance vessels, maintaining constant precapillary pressure through autoregulatory mechanisms. The impairment of the vasomotor tone and response during ischemia–reperfusion contributes to CMVO. The dense capillary network (approximately 2200 per square mm), which holds up to one-third of the myocardial blood volume, is particularly vulnerable to microcirculatory clogging, subsequently undergoing vasoconstriction, obstruction, and compression in the no-reflow phenomenon (Figure 1). In contrast, venules, with their thinner smooth muscle layers, primarily contribute to inflammatory processes by serving as adhesion sites for leukocytes and platelets during reperfusion injury [12,13].

**Figure 2:**
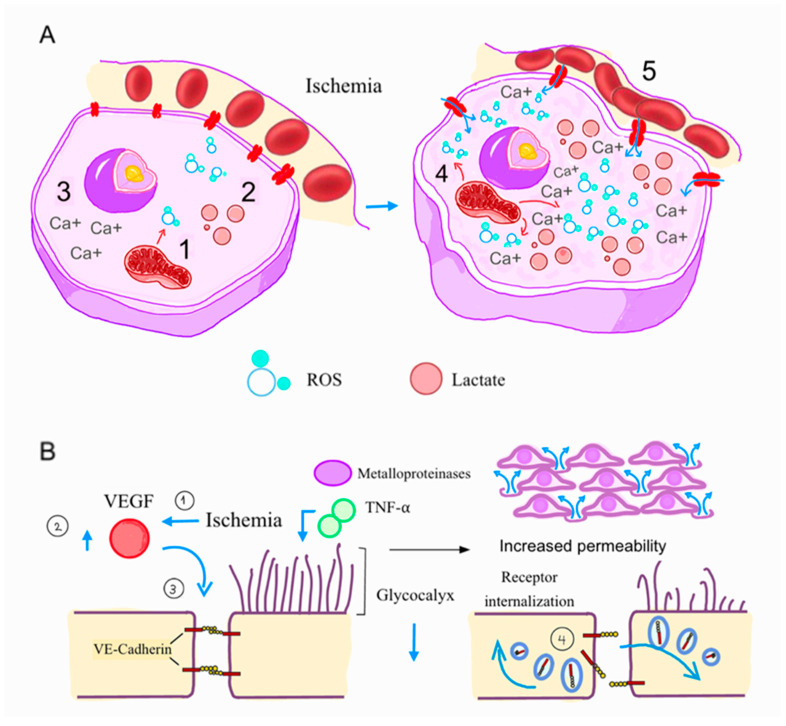
**Molecular pathways of microvascular dysfunction.** (**A**) Reduced aerobic metabolism causes ischemia, leading to cardiomyocyte death. These types of cell death are initiated by a series of intracellular disturbances, including impaired ion transport from ATP depletion and calcium overload (**3**), and the production of reactive oxygen species (ROS) by dysfunctional mitochondria (**1**). At the same time, the buildup of metabolic byproducts, such as lactate, occurs (**2**). These alterations lead to swelling of endothelial cells, luminal protrusion, as well as edema of cardiomyocytes (**4**) and interstitial fluid accumulation, culminating in microvascular obstruction (**5**). (**B**) Ischemia-induced factors, such as vascular endothelial growth factor (VEGF), promote the internalization of vascular endothelial VE-cadherin by endothelial cells, destabilizing intercellular junctions. The endothelial glycocalyx is prone to early degradation due to elevated levels of tumor necrosis factor-alpha (TNF-α) and matrix metalloproteinases. This degradation leads to increased vascular permeability, resulting in edema and inflammation.

***Myocardial ischemia:*** As classically described by Jennings and Reimer, the abrupt occurrence of ischemia resulting from the blockage of a primary coronary artery triggers various metabolic alterations. These include the halting of oxidative metabolism, reduction in creatine phosphate, a swift transition to anaerobic glycolysis, and a buildup of glycolytic byproducts like lactate and alpha-glycerol phosphate, along with the breakdown products of nucleotide pools in the affected tissue (Figure 2). As little as 15 to 20 s of ischemia can cause anaerobic glycolysis to take over as the sole source of energy, replacing oxidative phosphorylation and ATP. Most severely ischemic myocytes die within only 60 min, while less severely ischemic myocytes (subepicardial myocardium) survive for as long as six hours [14]. As ischemia persists, the detrimental effects of ischemia-related injuries become more pronounced, impacting the endothelial cells, cardiomyocytes, and interstitium [15].

***Endothelial cells, cardiomyocytes, and interstitium:*** The endothelium exhibits greater resistance to ischemia than cardiomyocytes. During myocardial ischemia, glycogen serves as the primary substrate for anaerobic glycolysis, leading to the rapid breakdown of glycogen stores. The lactate generated by anaerobic metabolism accumulates in cells and the interstitial space of ischemic tissue, along with other glycolytic metabolites, such as protons, inorganic phosphate, sugar phosphates, alpha-glycerol phosphate, glucose-6-phosphate, and glucose-1-phosphate. The buildup of these metabolic byproducts, particularly protons and lactate, eventually exceeds the capacity for their removal due to a lack of coronary washout, resulting in intracellular acidosis and increased osmotic gradients in both cells and the interstitium. This causes fluids to flow in, leading to cellular swelling and tissue edema [14,16].

Ionized calcium levels in the cytoplasm increase remarkably due to dysfunction of the ATP-dependent calcium extrusion and release from the endoplasmic reticulum, leading to the activation of cellular contractile elements. This rise is associated with the loss of contractile function, electrical uncoupling, and cytoskeletal and membrane destabilization due to activation of calcium-dependent proteases and phospholipases, leading to the degradation of cytoskeletal proteins and membrane phospholipids [17,18,19]. Furthermore, ischemia-related factors like vascular endothelial growth factor (VEGF) lead to alterations in cadherin-mediated cell–cell adhesion and junctions, destabilizing endothelial intercellular junctions and causing vascular permeability edema [20]. Additionally, VEGF activates nitric oxide synthase (eNOS), which increases vascular permeability (Figure 2) [20].

Elements of the cellular membrane, like the glycocalyx, also play fundamental roles in regulating vascular porosity. The endothelial glycocalyx is a highly hydrophilic, 0.5 µm gel-like layer composed of glycosaminoglycans, proteoglycans, and a carbohydrate-rich matrix, which is vital for preserving barrier integrity and regulating vascular permeability. This protective layer lines the endothelium, facilitating blood flow through capillaries, regulating vascular permeability, and mitigating leukocyte adhesion [21]. This structure, particularly the syndecans, heparan sulfates, and hyaluronan, suffers early degradation when under ischemia due to a direct damage caused by reactive oxygen species (ROS) and oxidized lipoproteins, while tumor necrosis factor-alpha (TNF-α) upregulates the expression of matrix metalloproteinases 2 and 9 (Figure 2) [22]. Conversely, nitric oxide protects the glycocalyx matrix from damage [23]. Previous anatomopathological analysis demonstrated that degradation of the endothelial glycocalyx compromises vascular barrier function, leading to heightened permeability, interstitial edema, and triggering inflammatory responses within the myocardium, enabling leukocyte and platelet adhesion to the capillary cell surface space [24,25,26].

During inflammation, platelets and neutrophil aggregates are formed and drawn to the capillaries and interstitial space. In the final common pathway, adhesion molecules on platelets are exposed, causing them to cluster around the damaged endothelial cells, neutrophils, and erythrocytes, creating a thrombotic complex that obstructs microcirculation. In addition to creating mechanical blockage, activated platelets shed microvesicles and apoptotic bodies, releasing various substances, including potent vasoconstrictors (thromboxane A2 and serotonin), pro-coagulants, adhesive proteins, and complement factors. Ultimately, these substances lead to endothelial destruction, encourage intracapillary coagulation, and stimulate chemotaxis and recruitment of leukocytes, further enhancing inflammation, angiogenesis, and apoptosis in the microvasculature [27].

Looking specifically at cardiomyocytes, the decrease in aerobic metabolism leads to cell death—either through necrosis, apoptosis, necroptosis, or pyroptosis—due to several changes. These include ion pumps failing because of ATP deficiency, acidosis, calcium overload, and the production of reactive oxygen species (ROS) by malfunctioning mitochondria [14].

***Distal embolization:*** Distal embolization plays a pivotal role in CMVO. It involves the mechanical and biochemical consequences of embolic debris composed of platelet-fibrin aggregates, atheromatous fragments, and cholesterol crystals. The phenomenon can arise spontaneously or iatrogenically during PCI due to instrumentation such as guidewire passage, balloon inflation, or stent deployment. Distal embolization happens more frequently in plaques containing large atheroma volumes, vulnerable plaques, as assessed by intracoronary and noninvasive coronary imaging [28]. In a similar vein, a connection exists between microembolization and pre-balloon dilation [29]. As microthrombi tend to accumulate in well-perfused and viable heart tissue, distal embolization affects salvageable myocardium. This contributes to CMVO through mechanical obstruction, increased vasoconstrictor tone, generation of prothrombotic substances, and favorable platelet aggregation due to robust inflammatory activation. In this environment, myocardial damage and mortality primarily manifest as necrosis, along with apoptosis, characterized by caspase-activated and caspase-mediated cell death [30]. The subsequent inflammatory response disrupts the contractile function of nearby surviving cardiomyocytes through signal transduction mechanisms that involve NO, TNF, sphingosine, ROS, and, ultimately, myofibrillar oxidation. Consequently, even minor quantities of microthrombi can create a localized reactive environment, triggering the release of substances that promote platelet aggregation, vasoconstriction, and endothelial dysfunction [30,31].

***Reperfusion-related injury and intramyocardial hemorrhage****:* Following reperfusion, myocardial injury is exacerbated by mechanisms such as cytoplasmic calcium overload, excessive ROS generation, and increased proteolytic enzyme activity, all of which contribute to structural damage of cell membranes and nuclear DNA. Simultaneously, ischemia–reperfusion triggers a strong inflammatory response marked by the influx of neutrophils, macrophages, and lymphocytes into the infarct area, exacerbating tissue damage and significantly contributing to CMVO. The earliest ultrastructural alterations occur in the subendocardial layer and progress toward the subepicardium [32]. One of the most severe manifestations of reperfusion-related injury is intramyocardial hemorrhage (IMH). Early autopsy findings from patients who experienced AMI and received thrombolytic treatment (intracoronary streptokinase) indicated that intramyocardial hemorrhage was exclusively seen after reperfusion, with none observed in nonperfused areas [33]. After prolonged ischemia and subsequent reperfusion, IMH often arises from endothelial cell necrosis, basement membrane disruption, and breakdown of microvascular integrity. Other contributing factors include microvascular damage caused by inflammation, the activation of the coagulation cascade, and the formation of intravascular thrombi, all of which promote the leakage of red blood cells into the myocardium tissue [34,35]. In addition to representing a serious type of ischemia–reperfusion injury, intramyocardial hemorrhage exacerbates CMVO by causing extracellular compression and other inflammatory response pathways. It is strongly correlated to adverse outcomes, even more than the extension of the infarction [36].

***Individual susceptibility:*** Multiple patient-specific characteristics may play a role in the pathogenesis of CMVO. These include genetic variations in the adenosine-induced vasodilatory response (such as the T19676C polymorphism in ADORA2A), variants in the region of VEGFA (significantly associated with microvascular dysfunction and angiogenic repair after ischemia in men), and CDKN-AS1 (also known as ANRIL, linked to atherosclerotic burden and microvascular remodeling) [37]. Sex-specific allelic variants, including MYH15 and NT5E, in males appear to be linked to a higher risk of CMVO. Furthermore, existing microvascular dysfunction in patients facing multiple cardiovascular risk factors may also contribute to an increased likelihood of developing CMVO [38,39].

## 3. Diagnosis

Microvascular obstruction can be evaluated using invasive methods, including conventional coronary angiography and novel catheter-based coronary physiology assessments. Nonetheless, noninvasive options like electrocardiograms, Coronary Magnetic Resonance (CMR), and angiography-derived coronary physiology indices are also available.

***Angiography:*** CMVO has typically been diagnosed and measured indirectly through conventional angiographic measures of no-reflow, such as the TIMI flow grade < 3, TIMI frame count, and myocardial blush grade. These measurements were largely used and validated as a prognostic tool after PCI [38]. Implementing these traditional criteria, the incidence of CMVO in patients suffering from AMI is about 30%, depending on the criteria and cohort [6]. However, this method does not properly represent tissue perfusion and microvascular function, lacking sensitivity when diagnosing more subtle cases of no-reflow. Although achieving an optimal final coronary angiographic result is associated with better prognosis after PCI, it correlates poorly with CMVO and coronary no-reflow assessed by CMR.

**Figure 3:**
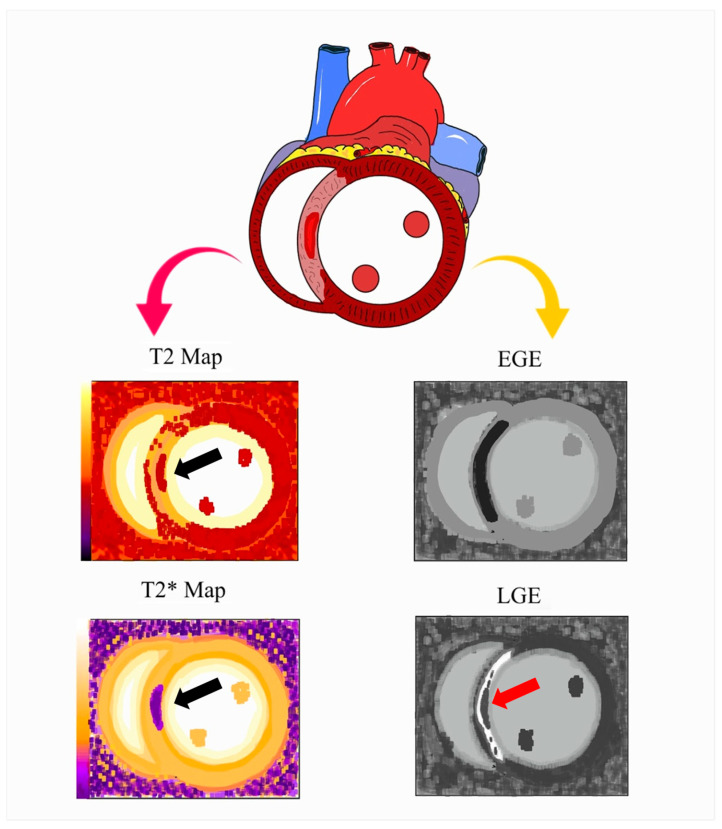
Schematic representation of coronary microvascular obstruction (CMVO) and intramyocardial hemorrhage (IMH) by CMR. The hypointense core on LGE represents CMVO (red arrow below) within the hyperenhanced infarcted region, indicating the absence of contrast uptake. It can also be identified during the first-pass perfusion phase (early hypoenhancement) (EGE). In cases of severe microvascular damage, erythrocyte extravasation into the myocardium results in IMH, which appears as a hypointense core on T2 and T2* mapping (black arrows).

***Cardiac Magnetic Resonance:*** CMR is the most sensitive method and the gold standard for detecting CMVO and no-reflow. Coronary no-reflow on CMR is shown as a lack of contrast uptake (a dark hypointense core in the area of hyperenhancement) during the first pass of the agent (early), or the lack of late gadolinium enhancement after 15 min of injection [40,41], with the latter showing better correlation to left ventricular remodeling and adverse cardiovascular events [42,43] and the former being a more sensitive finding. CMR has reported higher prevalences of CMVO, yielding rates as high as 57% in late-presenting STEMI [5]. When coronary microvascular injury following STEMI is particularly severe and vessel integrity is compromised, red blood cells may leak into the myocardium, characterizing IMH. The degradation of hemoglobin within the myocardium appears as a hypointense core on T2* imaging and T2* mapping, with the latter demonstrating higher sensitivity (Figure 3). CMR can accurately measure the infarct size, the amount of salvage myocardium, and the at-risk area.

***Physiology-based methods:*** Over the past three decades, the assessment of microcirculation has become possible through dedicated guidewires capable of measuring temperature and pressure. These devices allow calculation of several indices related to the functional status of the entire coronary vascular bed (i.e., epicardial vessels and microcirculation) at the time of the intervention.

To summarize, microvascular injury (and consequently CMVO) is marked by a decrease in coronary flow reserve as well as a rise in the hyperemic microvascular resistance index and the microcirculatory resistance index (IMR). IMR stands out as the most widely utilized parameter for assessing CMVO [44,45,46]. It evaluates microcirculation status without the effect of concomitant residual epicardial arterial narrowing. IMR is determined by multiplying the distal pressure by the mean transit time of 3 mL of room-temperature saline, which is measured using traditional thermodilution methods; consequently, the unit of IMR (U) is mmHg·s (1 mmHg = 0.133 kPa). Measurements are taken at rest and during steady-state hyperemia, which is induced by an intracoronary adenosine infusion. This index correlated with short- and long-term outcomes following a myocardial infarction, with values > 25 U being considered abnormal and >40 U being strongly associated with death and hospitalization for heart failure. IMR has even been proposed as a guiding parameter for adjunctive therapy during primary PCI [45,46]. Interestingly, even in the non-acute setting, changes in microvascular resistance are associated with myocardial injury, and post-PCI IMR is linked to periprocedural myocardial infarction following elective PCI [47].

Recent advancements in the understanding and application of computational fluid dynamics (CFD) in the coronary flow have enabled the development of angiography-derived indices of microcirculatory resistance, referred to as “angio-IMR”. This innovation eliminates the requirement for pressure-wire insertion and the use of adenosine [48,49]. These indices have recently been validated as an accurate and minimally invasive method to predict IMR and CMVO, as assessed by CMR, while also enhancing the risk stratification of MACE (Major Adverse Cardiac Event) in patients with STEMI [48,50].

## 4. Treatment (Figure 4)

### 4.1. Pharmacological Treatment with Antithrombotic Agents

The main reasons for administering antithrombotic agents after an acute myocardial infarction are to decrease thrombus burden, promote its breakdown, minimize distal embolization, and prevent the formation of new clots. Antiplatelets, anticoagulants, and thrombolytic agents have been tested as possible treatments for CMVO.

***Anticoagulants:*** In addition to antiplatelet therapy, anticoagulants play a critical role during primary PCI to prevent thrombotic complications. Unfractionated heparin (UFH) and bivalirudin are the two most widely used and studied options. The MATRIX trial compared bivalirudin to UFH in patients undergoing PCI and demonstrated no significant difference in the incidence of major cardiovascular events or net adverse clinical events [51]. However, more recent evidence suggests potential advantages of bivalirudin in specific clinical contexts. In an RCT with STEMI patients undergoing primary PCI, predominantly with radial access, the use of a basal dose of bivalirudin plus a high-dose post-PCI dose reduced the 30-day composite rate of all-cause mortality or major bleeding compared with heparin monotherapy, without an increase in ischemic events [52]. These findings have been confirmed in a subsequent meta-analysis [53]. Specifically regarding CMVO, bivalirudin was associated with a reduction in ischemia/reperfusion injury in STEMI patients as assessed by CMR, reducing the volume of MVO and intramyocardial hemorrhage compared to UFH [54].

***Oral antiplatelet agents:*** Aspirin is the most commonly used oral antiplatelet drug. It irreversibly inhibits the activity of the cyclooxygenase enzyme (COX) by specifically acetylating a serine residue in the active sites of both COX-1 and COX-2. COX-1 primarily synthesizes thromboxane A2 (TX-A2), which promotes platelet aggregation and vasoconstriction. Meanwhile, COX-2 diverts the arachidonic acid pathway towards the formation of prostaglandin I2 (PGI2), a potent inhibitor of platelet aggregation and a vasodilator. Lower doses of aspirin are used in coronary syndromes (75–150 mg) to inhibit COX-1, while only higher doses inhibit COX-2, but this is not standard in cardiovascular care. Oral thienopyridines (clopidogrel and prasugrel) selectively inhibit adenosine diphosphate (ADP) induced platelet aggregation through platelet P2Y12 receptor inhibition. Clopidogrel, while widely used, is less potent and subject to variable responses due to genetic and pharmacokinetic factors. Treatment with a potent oral P2Y12 inhibitor (prasugrel or ticagrelor), in addition to aspirin, is considered the standard of care for patients with MI due to the reduction in ischemic events [55,56]. Concerning CMVO, the treatment with potent P2Y12 inhibitors was associated with a smaller infarct size and lower microvascular obstruction incidence compared to clopidogrel [57]. Despite some conflicting studies, a strategy combining aspirin with ticagrelor demonstrated improved microvascular function in ACS patients compared to aspirin combined with clopidogrel [58,59,60].

***Parenteral P2Y12 inhibitors:*** Cangrelor is a fast-acting, intravenous, reversible P2Y12 inhibitor that may reduce infarct size and CMVO in patients with STEMI. Indeed, a large RCT linked it to fewer periprocedural ischemic events, including myocardial infarction, stent thrombosis, and ischemia-driven revascularization. However, compared to potent oral P2Y12 inhibitors like ticagrelor, there was no significant difference in myocardial perfusion indices between the two interventions [61,62]. The PITRI trial, a phase 2 randomized trial, demonstrated no benefit of cangrelor in infarct size and CMVO compared to placebo, on top of ticagrelor, despite a significant reduction in platelet reactivity [63]. Novel subcutaneous P2Y12 inhibitors (such as selatogrel) are also undergoing testing [64].

***Glycoprotein IIb/IIIa inhibitors:*** The glycoprotein IIb/IIIa receptor is the final common pathway for platelet aggregation, mediating inter-platelet adhesion and aggregation via fibrin. Its inhibition is one of the therapeutic targets for managing coronary thrombosis (e.g., abciximab, tirofiban, eptifibatide). However, this category of drugs has shown limited benefits in improving clinical outcomes, alongside a higher risk of bleeding, resulting in a class III recommendation in the latest guidelines for routine use. The current guidelines indicate its use for patients with significant intracoronary thrombotic load and no-reflow [65]. Despite that, the REVERSE-FLOW trial directly addressed the efficacy of a bailout GP IIb/IIIa inhibitor strategy in AMI patients with angiographic CMVO. In this trial, this class of medications failed to reduce the primary endpoint of infarct size. Moreover, a decrease in CMR-derived CMVO was noted, accompanied by an increase in non-fatal bleeding events [66,67] and no net clinical benefit. Novel subcutaneous glycoprotein inhibitors (e.g., zalunfiban) are currently under investigation. In a phase 2 trial, higher doses of zalunfiban, given before arterial access, correlated with improved coronary and myocardial perfusion and reduced thrombus burden seen at the initial angiogram in patients with STEMI receiving primary PCI [68].

### 4.2. PCI Technique and Adjunctive Devices

***Delayed or deferred stent implantation strategy:*** This strategy has been proposed to reduce the risk of no-reflow in patients with ST-segment elevation myocardial infarction (STEMI) and has been extensively studied, with the primary rationale being the reduction in distal embolization. However, evidence from multiple randomized trials and meta-analyses—such as DANAMI 3-DEFER-STEMI, MIMI, SUPER-MIMI, and INNOVATION—showed that, although safe, there was no benefit of routine delayed stent implantation in terms of hard endpoints such as death, myocardial infarction, or repeated revascularization. Instead, it reduces angiographic no-reflow and improves surrogates, like myocardial blush grade [69,70,71,72,73,74]. Sub-studies using CMR showed no difference in final infarct size, myocardial salvage, or CMVO [75]. However, since the benefit in these surrogate outcomes seems more pronounced in certain patients, especially those with high thrombus burden, long lesions, delayed STEMI presentation, or suboptimal TIMI flow after thrombus aspiration, a deferred stenting approach may still be safe and potentially beneficial [73,76].

***Mechanical embolization protection devices****:* Using embolic protection devices to prevent plaque and thrombus from embolizing from the coronaries has a solid rationale for protecting against CMVO during PCI. Several devices, such as distal coronary filters, have been tested. In the case of saphenous vein graft angioplasty, a distal filter has been shown to lower the combined primary endpoint of death, MI, emergency bypass, and target lesion revascularization at 30 days [77]. However, in modern PCI, although improving angiographic surrogates of CMVO, embolic protection devices have not shown benefit in reducing MACE in either native or saphenous vein graft PCI [78,79].

***Thrombectomy****:* Mechanical aspiration devices have long been employed in STEMI procedures to reduce the thrombus burden. Thrombectomy techniques can be classified as either manual or mechanical. Manual thrombectomy can be achieved with a series of devices that share a common feature: They eliminate thrombus by creating a vacuum using a syringe linked to the proximal hub. An early, single-center trial has demonstrated improved myocardial blush grade and ST-segment resolution with an early signal of reduction in cardiac mortality [80]. However, in this context, two larger RCTs have been published. The TOTAL and the TASTE trials tested a similar strategy of upfront routine manual thrombectomy. Both trials failed to demonstrate significant differences in clinical outcomes when comparing PCI combined with routine manual thrombectomy versus PCI alone. The TOTAL trial, which randomized more than 10,000 patients, revealed an increased stroke risk in patients who received thrombectomy compared to those treated with PCI alone (HR 2.06; 95% CI 1.13–3.75) [81,82]. Conversely, a meta-analysis incorporating data from the two aforementioned RCTs, involving a total of more than 19,000 STEMI patients, showed no improvement in clinical outcomes. However, in the high thrombus group, there was a reduction in cardiovascular deaths and an increase in cerebrovascular events [83]. Mechanical thrombectomy, on the other hand, relies on an external machine as its energy source to perform aspiration. The CHEETAH study demonstrated, in 400 patients, encouraging results regarding safety, featuring high rates of thrombus removal, restored blood flow, and enhanced myocardial perfusion on final angiography [84].

**Sonothrombolysis**: The microbubble cavitation, generated by the delivery of high mechanical index ultrasound pulses by a standard transthoracic transducer within the thrombus, produces shear forces that can dissolve both epicardial and microvascular thrombi. It is hypothesized to stimulate the release of nitric oxide from endothelial cells and red blood cells. This concept was initially evaluated in a study involving 100 STEMI patients who were randomized to receive sonothrombolysis prior to and following either primary PCI or standard PCI. Added to PCI, sonothrombolysis improves recanalization rates and reduces infarct size, leading to lasting improvements in systolic function following STEMI [85]. Additionally, a sub-study from the same group has demonstrated improved CMVO and myocardial dynamics [86]. Despite these merits, further research is still needed concerning its implementation as a regular strategy for treating acute MI.

***Mechanical unloading:*** Mechanical ventricular unloading refers to any device-based intervention that decreases the external work of the heart and is generally associated with decreased wall stress (especially during diastole) and decreased myocardial oxygen consumption. Preclinical studies showed that mechanically unloading the left ventricle during the acute phase of a myocardial infarction, before reperfusion, reduces the size of the infarct, preserves mitochondrial structure and function, improves myocardial energy utilization, and prevents subsequent heart failure [87,88]. The DTU-STEMI (Door-To-Unload in STEMI) was the first pilot study to demonstrate the feasibility and safety of mechanical unloading with a percutaneous transaortic pump and delayed reperfusion in STEMI patients [88,89]. Following the publication of the pilot study, a per-protocol analysis showed that LV unloading for 30 min before reperfusion significantly reduced the ratio between infarct size and area at risk compared to LV unloading and immediate reperfusion [90]. The STEMI-DTU study (NCT03947619) is a larger randomized trial designed to compare LV unloading and delayed reperfusion with standard care in patients with STEMI. This study has completed enrollment, and the results are expected to be available by the end of 2025.

## 5. Enhancing Microvascular Function and Reducing Ischemia–Reperfusion Injury

### 5.1. Anti-Inflammatory Drugs

Ischemia–reperfusion injury initiates an inflammatory response aimed at clearing debris from the infarcted area. However, an overly intense inflammatory response may exacerbate interstitial and myocardial edema, leading to CMVO.

***Statins:*** Statins have pleiotropic effects, enhancing endothelial function, promoting dilation of coronary microvasculature, and providing anti-inflammatory and antithrombotic benefits [91]. This may explain the beneficial impacts of high-dose statin treatment in the acute phase of myocardial infarction [92,93].

***IL-6 inhibitor****:* Tocilizumab, an IL-6 antagonist, has emerged as a promising agent for reducing the inflammatory burden encountered during acute MI since IL-6 is a pro-inflammatory cytokine implicated in endothelial injury, inflammatory cell recruitment, and microthrombi formation. One placebo-controlled RCT showed, in 199 STEMI patients undergoing PCI, that tocilizumab increases the primary endpoint of the myocardial salvage index and reduces CMVO, as assessed by CMR [94]. Mechanistic insights from a sub-study demonstrated that tocilizumab led to a rapid and pronounced reduction in circulating neutrophil counts and attenuated neutrophil function [95].

***Other anti-inflammatory agents:*** Triiodothyronine (T3) has been shown to activate the Reperfusion Injury Salvage Kinase (RISK) pathway and consequently improve mitochondrial function, microvascular integrity, and reduce edema, leading to infarct size reduction in rats [96] and promising results in humans [97]. Cyclosporine A inhibits the opening of mitochondrial transition pore (mPTP), preventing mitochondrial swelling and necrotic cell death. Despite that, cyclosporine did not improve myocardial reperfusion injury, infarct size, or left ventricular function [98].

***FDY-5301:*** Alongside the previously discussed therapies and ongoing trials, FDY-5301 represents a novel, investigational molecule aimed at downregulating the pathological inflammatory response associated with both acute and chronic reperfusion injuries. Comprising sodium iodide, this agent acts as a catalytic anti-peroxidant, converting hydrogen peroxide (a potent Reactive Oxygen Species—ROS) into water and oxygen, potentially neutralizing the harmful effects of ROS that occur during reperfusion. A previous phase 2 study with 120 STEMI patients demonstrated that administering intravenous FDY-5301 just before primary PCI in acute STEMI patients is feasible and safe, potentially reducing the final infarct size [99]. The IOCYTE-AMI 3 trial plans to include 2300 subjects to evaluate the impact of FDY-5301 on cardiovascular death and acute heart failure occurrences in patients with anterior STEMI who are receiving PCI.

***Supersaturated Oxygen (SSO_2_):*** This therapy involves localized infusion of supersaturated oxygen (SSO_2_) into the infarct-related coronary artery. SSO_2_ has previously been demonstrated to reduce endothelial cell edema, induce capillary vasodilatation, limit infarct size, and improve left ventricular remodeling in preclinical models [100,101,102]. The AMIHOT I trial involved 269 patients with acute anterior or extensive inferior myocardial infarctions who were treated with either primary or rescue PCI [93]. These patients were randomly assigned to receive either 90 min of intracoronary supersaturated oxygen (SSO_2_) therapy or normoxemic blood autoreperfusion. Results demonstrated that, although the primary endpoint of MACE did not differ between groups, among patients with anterior MI reperfused within six hours, those randomized to SSO_2_ exhibited a greater improvement in regional wall motion, smaller infarct size, and improved ST-segment resolution. Similar findings were obtained from AMIHOT-II [103], leading to regulatory approvals for clinical use in Europe and the United States. One hypothesized mechanism of action for SSO_2_ therapy is its beneficial effect on microvascular function. A pooled analysis of individual patient data from 9 studies revealed, for the first time, that post-PCI SSO_2_ infusion is also associated with reduced MVO [104]. From this non-randomized, multivariable-adjusted comparison of the outcomes of two SSO_2_ therapy studies vs. seven control studies, SSO_2_ infusion was associated with a significant reduction in the extent of MVO, and a trend of reduction in the presence of any MVO as assessed by late gadolinium enhancement CMR scan. The recently announced HOT-AAMI trial aims to be the first trial powered to determine whether SSO_2_ therapy, administered immediately post-PCI, improves death and heart failure outcomes in patients with anterior STEMI [105].

***Ischemic preconditioning, postconditioning, and remote preconditioning:*** This strategy encompasses a range of cardioprotective therapies, pharmacological and non-pharmacological, applied before ischemia or during reperfusion. These interventions activate intracellular signaling pathways involving survival protein kinase (activation of ERK1/2 and PI3K-Akt) and antiapoptotic (Bcl-2 and BAX) pathways, protein kinases C and G activation, production of nitric oxide, opening of ATP-sensitive potassium channels (KATP), and blockade of the mitochondrial pores [106]. This may lead to a decrease in both necrotic and apoptotic cell death, reducing the extent of no-reflow and the size of the infarct in multiple animal models [107]. Short episodes of intermittent ischemia during the initial phase of reperfusion might lead to a decrease in infarct size. Importantly, a small, randomized trial indicated that postconditioning correlates with a 36% reduction in infarct size [108]. Nonetheless, a large RCT did not show any advantages of ischemic conditioning over standard therapy regarding the primary composite endpoint, which includes death from any cause and hospitalization due to heart failure [109]. Remote ischemic conditioning, typically achieved by intermittent inflation and deflation of a blood pressure cuff on a limb, could also have a potential benefit in decreasing CMVO [110]. This technique has been shown to have no clinical benefit in a large clinical trial [111,112].

***Myocardial cooling:*** In animal models of STEMI, inducing hypothermia effectively reduces reperfusion injury. In animal models, hypothermia protects against coronary no-reflow and triggers various cardioprotective signaling pathways [113]. Despite the positive outcomes in models, these results have not been successfully replicated in humans. Aiming to make the application of hypothermia more practical, a method has been created that selectively cools the infarct region while utilizing standard interventional tools. A RCT named the EURO-ICE trial (NCT03447834) plans to enroll 200 anterior STEMI patients with TIMI flow 0–1 as a proof-of-concept study to evaluate the benefits of selective intracoronary cooling [114].

**Pressure-controlled intermittent coronary sinus occlusion (PiCSO):** This method involves intermittently increasing the pressure in the cardiac venous outflow tract using a balloon-tipped catheter positioned in the coronary sinus (CS). The rationale is centered on three mechanisms. First, a transient increase in CS pressure leads to a redistribution of blood from remote myocardium to the border zone of the ischemic myocardium. Second, during the deflation of the CS balloon, the pressure drop facilitates the washout of interstitial fluids (edema) and the removal of thrombotic debris and toxic metabolites. Lastly, the pulsatile variation in the CS pressure produces shear stress that activates the endothelium and the pericytes of the myocardial veins, favoring the upregulation of cardioprotective, pro-angiogenic, and regenerative pathways. This has been demonstrated in animal models [115,116]. Early feasibility studies in humans have been published and suggest a reduction in infarct size and microvascular function, as well as an improvement in anginal scores [117]. Conversely, a large RCT involving 145 anterior STEMI patients comparing a combined PCI + PiCSO implantation strategy to standard primary PCI failed to demonstrate a reduction in infarct size, CMVO, or intramyocardial hemorrhage. [118].

### 5.2. Pharmacological Treatment of Established Non-Reflow During PCI

**Vasodilators:** As previously mentioned, vasoconstriction significantly contributes to CMVO and is regulated by various receptors in epicardial arteries and microvessels, including α1-adrenergic and α2-adrenergic receptors. Therefore, multiple pharmacological strategies are aimed at intervening in this path.

***Adenosine:*** Adenosine, a key endogenous purine nucleoside, is an extracellular signaling molecule that plays a crucial role in maintaining coronary microvascular tone, particularly during ischemia. Its effects are primarily mediated through the adenosine A2A receptor, which promotes vasodilation on both endothelial and smooth muscle cells, and, by association, the production of NO and the activation of ATP-sensitive and voltage-dependent potassium channels (KATP) [119]. Additionally, adenosine has multiple effects, such as anti-inflammatory and antiplatelet activation properties. It plays a role in promoting ischemic preconditioning, limiting reperfusion injury, and inhibiting cardiomyocyte apoptosis [120,121]. The mechanism of action may also include the suppression of neutrophil migration, reduction in superoxide production, and inhibition of endothelin release from the coronary bed. Results of RCTs studying the administration of adenosine during primary PCI have yielded mixed results [122,123,124,125,126]. The differing outcomes may be attributed to significant heterogeneity among therapeutic schemes, routes of administration, and dosages used. Of note, in the REOPEN-AMI Trial, 240 STEMI patients with TIMI flow 0–1 were randomly allocated 1:1:1 to receive either adenosine (120 µg as a fast bolus, followed by 2 mg as a slow bolus), nitroprusside, or saline given distal to the occluded site and after thrombus aspiration [123]. It was seen that intracoronary adenosine was associated with an improvement in CMVO (as assessed by angiographic electrocardiographic criteria) and a smaller infarct area. This benefit also led to a reduction in MACE and left ventricular remodeling in 1 year [125]. Still, another 3-arm RCT with a similar size, the REFLO-STEMI trial [127], randomized STEMI patients to a high dose (2–3 mg total) of intracoronary adenosine, nitroprusside, or standard care immediately after thrombectomy and repeated after stenting. This study found that neither adenosine nor nitroprusside decreased the infarct area or CMVO, as assessed by CMR. Furthermore, adenosine was associated with detrimental cardiac effects and worse clinical outcomes. One hypothesis for these negative results is the adenosine-induced coronary steal [128]. Still, a meta-analysis published by Bulluck et al. included 13 RCTs (a total of 4273 STEMI patients) that demonstrated a reduction in the incidence of heart failure and coronary no-reflow [129]. Other meta-analyses, however, showed an increased risk of advanced atrioventricular block or ventricular arrhythmias, especially in patients who experienced prolonged ischemia [130].

***Nitroprusside:*** Nitroprusside is a nitric oxide donor that acts independently of intracellular metabolism. It has been tested in at least two RCTs [123,127] and did not show improvement in CMVO compared to placebo or verapamil [131]. It is noteworthy that since the microvasculature cannot metabolize nitroglycerin to NO, nitroglycerin is not a valid option in this scenario.

***Nicorandil:*** Nicorandil is a hybrid anti-anginal agent with both KATP-activating properties and nitrate-like effects. As such, nicorandil may prevent reperfusion injury by blocking mitochondrial permeability transition pore opening, thus reducing cardiomyocyte death, mimicking ischemic preconditioning. Nicorandil also acts as a nitric oxide donor, causing coronary vasodilation. Administered prior to primary PCI in STEMI patients, it has been shown to enhance angiographic measures of myocardial reperfusion, elevate left ventricular ejection fraction, and decrease infarct size when compared to placebo [132]. The drug has been shown to reduce microvascular dysfunction after PCI for stable angina [133]. A meta-analysis that included 18 RCTs and 2055 STEMI patients undergoing primary PCI found that administering nicorandil during the procedure improved coronary blood flow and cardiac systolic function while also improving prognosis [134].

***Calcium channel blockers:*** Calcium channel blockers such as verapamil, diltiazem, and nicardipine work by blocking calcium entry through calcium channels in vascular smooth muscle and myocardial cells. This leads to coronary vasodilation, particularly in microcirculation, alleviates vasospasm, reduces myocardial contractility, and lowers myocardial oxygen demand [135]. Potential benefits such as decreased microvascular spasm and lowered heart rate and blood pressure could lead to a smaller infarct size. A previous RCT randomized STEMI patients to either verapamil, adenosine, or placebo [136]. In comparison to placebo, verapamil enhanced coronary flow equally as adenosine. However, compared to adenosine, verapamil caused a higher incidence of transient heart block. Compared to nitroprusside after primary PCI, verapamil significantly improved MVO with fewer adverse events [131].

***Epinephrine:*** Epinephrine, primarily known for its vasopressor effects, also acts as a *β2*-adrenergic receptor agonist, promoting coronary vasodilatation. The COAR trial randomized 201 normotensive patients with acute coronary syndromes and PCI-related no-reflow to either intracoronary epinephrine or adenosine. The epinephrine group showed a significant improvement in coronary flow, as measured by the predefined primary endpoint (higher frequency of TIMI III flow, lower TIMI frame count, and better grade III myocardial function blush) [137].

***Papaverine***: Papaverine is a true alkaloid naturally derived from opium, sourced from the opium poppy (Papaver somniferum). It is established as one of the main vasodilator drugs used to treat erectile dysfunction, intestinal/gastric spasms, bronchospasm, and especially for peripheral and coronary arterial vasodilation. It is highly bioavailable (80%), has a high protein binding rate (~90%), and a half-life of 90–120 min. It acts through the inhibition of cAMP and cGMP phosphodiesterase, leading to increased tissue levels of cAMP and cGMP and a reduction in the influx of calcium ions (Ca^2+^) into the cytoplasm. The elevation of cGMP results in increased activity of protein kinase G (PKG), which reduces the influx of Ca^2+^ and increases the efflux of potassium (K+), culminating in the hyperpolarization of the cells. This rise in cAMP and cGMP, along with the decrease in Ca^2+^ levels, leads to reduced activity of myosin light chain kinase (MLCK), which is responsible for activating myosin in smooth muscles [138,139,140,141]. Concurrently, they enhance the action of myosin light chain phosphatase (MLCP), which dephosphorylates the myosin molecule, promoting smooth muscle cell relaxation and vasodilation. A small, non-randomized study demonstrated that intracoronary use of papaverine resulted in a significant improvement in perfusion with a 56% decrease in mean TIMI frame count (i.e., the number of cineframes required for the contrast medium to reach standardized distal landmarks of a coronary artery), indicating an attenuation of angiographic no-reflow [142].

## 6. Conclusions

The current standard of care in the setting of acute STEMI includes primary PCI, antiplatelet therapy, beta-blockers, statins, and ACEi/ARBs. One-year mortality rates following STEMI are approximately 7% [143], and the composite rate of mortality and heart failure hospitalization is 13% [144]. Accordingly, there is still an urgent need for improved therapies. While a wide range of viable therapeutic targets and approaches to reduce infarct size by targeting post-PCI CMVO and myocardial hemorrhage have been reviewed herein (Table 1), limited evidence from RCTs is available, and none has been proven to improve outcomes. Yet, numerous studies have consistently demonstrated a strong correlation between MVO, infarct size, and adverse clinical outcomes such as all-cause mortality and heart failure hospitalization within the first year. Among the many tested approaches, only intracoronary SSO_2_, delivered immediately following primary PCI, has been shown to reduce infarct size and to receive regulatory approval for this indication; studies to confirm improved clinical outcomes with this therapy are underway (ClinicalTrials.gov ID NCT06438315). Given the complex and multifaceted nature of no-reflow pathophysiology, as well as the difficulties in accurately diagnosing microvascular dysfunction, an optimal treatment strategy for MVO remains, thus far, elusive. Progress will depend on a precise understanding of coronary microcirculation function and the mechanisms by which emerging therapies aim to restore microcirculatory integrity.

## Figures and Tables

**Figure 4:**
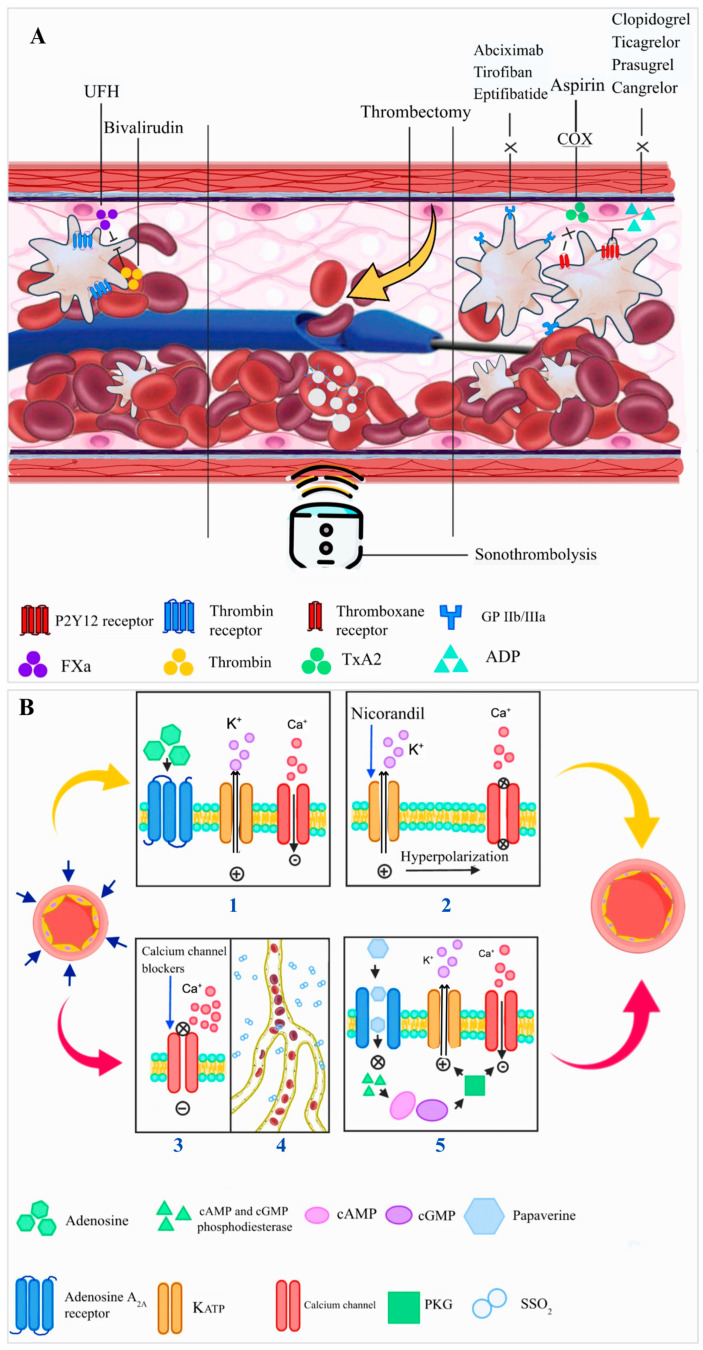
Interventions for Coronary Microvascular Obstruction (**A**) Pharmacological and mechanical Treatment (**B**) Vasodilators. (**1**) Adenosine has a direct vasodilatory effect through activation of the adenosine A2A receptor on endothelial cells. The mechanism of action involve the opening of (KATP), (**2**) Nicorandil exerts its vasodilatory effects through a dual mechanism: it acts as both a nitric oxide (NO) donor and an opener of KATP, the opening of KATP leads to an efflux of potassium ions, resulting in membrane hyperpolarization and further smooth muscle relaxation. (**3**) Calcium channel blockers exert their pharmacologic effects by inhibiting the influx of calcium ions through calcium channels in vascular smooth muscle and myocardial cells. This inhibition reduces intracellular calcium concentration, leading to vascular smooth muscle relaxation and coronary vasodilation, with a pronounced effect on the coronary microcirculation. (**4**) Supersaturated oxygen induces a state of hyperoxia that enhances endothelial nitric oxide synthase (eNOS) activity, resulting in increased NO production. NO subsequently activates soluble guanylate cyclase in vascular smooth muscle cells, leading to elevated intracellular levels of cGMP, which promotes vasodilation through smooth muscle relaxation. (**5**) Papaverine exerts its vasodilatory effects primarily through the non-selective inhibition of phosphodiesterases responsible for the degradation of cAMP and cGMP. This inhibition leads to elevated intracellular levels of cAMP and cGMP. The increase in cGMP activates PKG, which reduces cytosolic calcium (Ca^2+^) influx and enhances potassium (K^+^) efflux, resulting in membrane hyperpolarization and further inhibition of calcium entry into the cell, leading to smooth muscle relaxation and vasodilation. ADP, adenosine diphosphate; cAMP, cyclic adenosine monophosphate; cGMP, cyclic guanosine monophosphate; COX, cyclooxygenase enzyme; FXa, factor Xa; GP IIb/IIIa, glycoprotein IIb/IIIa; KATP, ATP-sensitive potassium channels; PKG, protein kinase G; SSO_2_, supersaturated oxygen; TxA2, thromboxane A2; UFN, Unfractionated heparin.

**Table 1:** Interventions for Coronary microvascular obstruction (CMVO).

Intervention	Mechanism	Evidence Summary
Antithrombotic Therapy		
Anticoagulants (UFH, Bivalirudin)	Inhibit thrombin activity and Factor Xa; reduce thrombus propagation and embolization risk during PCI.	Mixed results from RCT. Bivalirudin reduces major bleeding compared to UFH, with mixed effects on thrombotic outcomes. May reduce CMVO [51,52,53,54].
Oral P2Y12 Inhibitors (Ticagrelor, Prasugrel)	Inhibit ADP-mediated platelet aggregation by blocking P2Y12 receptor; enhance platelet inhibition vs. clopidogrel.	RCTs demonstrated ischemic benefit of potent inhibitors compared to clopidogrel and is recommended by guidelines. Conflicting evidence regarding CMVO assessed by CMR [55,56,58,60].
Parenteral P2Y12 Inhibitors (Cangrelor)	Reversibly blocks P2Y12 receptor; rapid onset/offset allows periprocedural platelet inhibition.	PITRI trial: no additional CMVO benefit on top of ticagrelor [63].
GP IIb/IIIa Inhibitors (abciximab, tirofiban, eptifibatide)	Block fibrinogen binding and final common pathway of platelet aggregation; used in bailout no-reflow scenarios.	REVERSE-FLOW: reduced MVO but did not decrease infarct size; increases risk of non-fatal bleeding events [66].
Inflammation and Endothelial Protection		
Statins	Stabilize endothelium via pleiotropic effects; reduce oxidative stress, inflammation, and preserve glycocalyx integrity.	Meta-analyses and RCTs show lower MVO incidence and preserved LV function with high-dose statins [92,93].
IL-6 Inhibitor (Tocilizumab)	Blocks IL-6 receptor, reducing systemic and local inflammation; modulates infarct healing and limits edema.	ASSAIL-MI: Small RCT showed an increase in myocardial salvage and reduction in CMVO [95].
Device-Based Mechanical Strategies		
Supersaturated Oxygen Therapy (SSO_2_)	Delivers high-dissolved O_2_ post-PCI; reduces capillary edema and enhances microvascular perfusion.	AMIHOT I/II: reduced infarct size and CMVO [102,104].
Thrombectomy (Manual/Mechanical)	Physically removes thrombus from culprit vessel; targets macro- and microembolization.	Manual: TOTAL/TASTE: no MACE benefit; not routinely recommended. Mechanical: CHEETAH trial: improved flow in high thrombus burden [81,82,83,84].
Sonothrombolysis	Microbubble-induced thrombus disruption via ultrasound cavitation; enhances clot resolution.	Small RCT enhanced recanalization and reduced infarct size [85].
Mechanical Unloading	Reduces myocardial oxygen demand pre-reperfusion; limits infarct size and MVO.	DTU-STEMI: delayed reperfusion after LV unloading reduced infarct size/area at risk [90]. A larger RCT finished enrolling. (NCT03947619).
Pressure-controlled Coronary Sinus Occlusion (PiCSO)	Increases venous pressure cyclically to redistribute flow, reduce edema, and activate endothelial recovery.	Failed to demonstrate a reduction in infarct size, CMVO, or intramyocardial hemorrhage [117,118].
Pharmacological established no-reflow treatment		
Papaverine	Inhibits phosphodiesterase → ↑cAMP/cGMP → smooth muscle relaxation; potent microvascular vasodilator.	Small study: 56% decrease in TIMI frame count [142].
Adenosine	Activates A2A receptor → vasodilation; also inhibits neutrophil adhesion and ROS production.	Heterogenous results. Intracoronary moderate–high dose of adenosine may improve CMVO and LVEF. Caution with advanced heart block and hemodynamic effects. Evidence from RCTs [123,126].
Nicorandil	Dual KATP channel opener and NO donor; preconditions myocardium and reduces microvascular resistance.	Improved coronary blood flow and cardiac systolic function. Also improved prognosis [132,133,134].
Calcium Channel Blockers	Inhibit L-type Ca^2+^ channels in smooth muscle; attenuate vasospasm and improve flow.	Compared to placebo, verapamil improved coronary flow. Compared to adenosine, verapamil also caused a higher incidence of transient heart block. Compared to nitroprusside, verapamil resulted in significant improvements in CMVO, with fewer adverse events [131,136].
Epinephrine	Stimulates β2-receptors on microvasculature → vasodilation; used in refractory no-reflow.	COAR trial: improved angiographic criteria of coronary no-reflow [137].
Nitroprusside	Releases NO directly; dilates both arterial and venous vasculature, including microvessels.	Did not show benefits compared to verapamil [123,127,131].
